# Characterization of novel alternative splicing sites in human telomerase reverse transcriptase (*hTERT*): analysis of expression and mutual correlation in mRNA isoforms from normal and tumour tissues

**DOI:** 10.1186/1471-2199-7-26

**Published:** 2006-08-29

**Authors:** Stein Sæbøe-Larssen, Ellen Fossberg, Gustav Gaudernack

**Affiliations:** 1 Section for Immunotherapy, Department of Immunology, The Norwegian Radium Hospital, Cancer Research Institute, University of Oslo, N-0310 Oslo, Norway

## Abstract

**Background:**

Human telomerase reverse transcriptase (hTERT) is a key component for synthesis and maintenance of telomeres on chromosome ends and is required for the continued proliferation of cells. Estimation of *hTERT *expression therefore has broad relevance in oncology and stem cell research. Several splicing variants of *hTERT *have been described whose regulated expression contributes to the control of telomerase activity. Knowledge of the different *hTERT *mRNA isoforms and the ability to distinguish between them is an important issue when evaluating telomerase expression.

**Results:**

By establishing cDNA-clone panels from lung and colon tissues, we could map *hTERT *clones individually for differences in DNA sequence. This made possible the identification of novel alternatively spliced sites as well as analysis of their frequency and mutual correlation in mRNA isoforms. Ten different alternatively spliced sites were detected, of which six were novel sites resulting from alternative splicing of intron 2 or 14. The majority of *hTERT *cDNA clones from normal and tumour lung and colon tissues encoded truncated proteins ending close after exon 2 or 6.

**Conclusion:**

The increased complexity in telomerase expression revealed here has implications for our understanding of telomerase regulation and for the choice of suitable methods for addressing *hTERT *expression.

## Background

The ends of eukaryotic chromosomes are usually capped by telomeres which consist of repeated copies of a short DNA sequence and several associated proteins [reviewed in [1]]. The telomeres protect the chromosomes from damage and degradation and from being fused together by DNA repair mechanisms and are essential for genomic integrity and cell viability. Since the conventional DNA replication process is unable to completely synthesize chromosome ends, proliferating cells lacking de novo synthesis of telomeric DNA will eventually lose their telomeres and enter a growth-arrest state called replicative senescence. The continued proliferation of eukaryotic cells is ensured by the telomerase enzyme which maintains and synthesizes telomeric repeats on to chromosome ends [2,3]. In humans, telomerase activity has been detected in many highly proliferative cells and tissues, such as early stage embryos, reproductive tissues in testis and ovary, stem cells, fibroblasts and activated lymphocytes [reviewed in [4]]. Most somatic tissues contain undetectable levels of telomerase activity, but restoration of telomerase activity is required for immortalization and continued growth of cancer cells. Thus, regulation of telomerase activity has important implications for many developmental processes including cell proliferation, differentiation, ageing and tumorigenesis.

The human telomerase holoenzyme is composed of two core subunits, the telomerase RNA component hTERC [5], which contains a template for telomere elongation, and the telomerase reverse transcriptase catalytic subunit hTERT [6-9]. While hTERC is widely expressed in human tissues irrespective of telomerase status, the expression of normal full-length *hTERT *correlates well with telomerase activity and seems to be the rate-limiting factor for telomerase activity in human cells [7,8,10-13]. The *hTERT *gene consists of 16 exons and spans ~37 kb of genomic DNA, of which ~33 kb is intronic sequences and the remaining ~4 kb corresponds to the *hTERT *mRNA transcript [14]. Since processing of *hTERT *pre-mRNA also yields non-functional alternatively spliced products, the correlation between *hTERT *gene expression and telomerase activity is complicated. To date, seven alternatively spliced sites (ASPSs) in the *hTERT *mRNA have been described [9,14,15]. Two ASPSs, α-deletion and γ-deletion, result from in-frame deletions of exonic sequences in exon 6 and 11, respectively. The α-deletion isoform appear to be a dominant inhibitor of telomerase activity when over-expressed [16,17]. The remaining ASPSs represent exonic deletions and/or insertion of intronic sequences that cause frame shift and premature termination of the open reading frame (ORF). Alternative splicing of *hTERT *has implications for the regulation of telomerase activity [10,18-20]. In particular, telomerase is down-regulated in many tissues by a shift to β-deletion splicing mode, in which exons 7 and 8 are deleted. Alternative splicing reconciles many of the inconsistencies observed between *hTERT *mRNA levels and lack of telomerase activity, but telomerase-negative cells frequently contain *hTERT *mRNA of which a fraction apparently is normal full-length [11,13,21,22]. It has been suggested that this may be due to downstream regulatory mechanisms, such as inhibitory factors or post-translational modification of the hTERT protein. It is also possible that the putative full-length *hTERT *mRNA in such cases contained unknown ASPSs, which consequently were not screened for. Here we report the characterization of six novel *hTERT *ASPSs detected in primary tissues from lung and colon that may be important for the regulation of telomerase activity in human cells.

## Results

### Characterization of *hTERT *ASPSs

**Figure 1:**
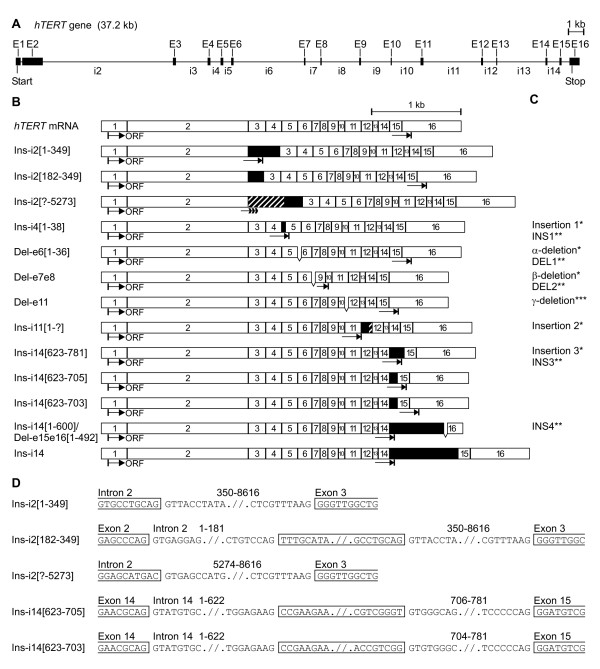
**Genomic organization and ASPSs of the *hTERT *gene**. (A) Exon/intron organization of the *hTERT *gene assembled from [Genbank:AF128893] and [GenBank:AF128894] [14]. Exons are shown as black boxes with numbering above. Start/Stop indicates the beginning and end of the ORF. (B) Schematic drawing of *hTERT *mRNA ASPSs. Exons are shown as open boxes with numbering inside. Black boxes represent intronic sequences and are cross-hatched to indicate uncharacterised 5' or 3' splice site. The ORF is indicated below each ASPS by arrows. The *hTERT *ASPSs have been assigned descriptive names: Ins-i, insertion of intronic sequence; Del-e, deletion of exonic sequence; followed by intron/exon number and a range of nucleotides in brackets when the insertion/deletion involves part of an intron/exon, respectively. (C) Formerly used designations: *[9], **[14], ***[15]. (D) Sequence of donor and acceptor splice sites. Unspliced sequence is boxed. Range of spliced nucleotides numbered from the first nucleotide of each intron is shown above the sequence.

To identify novel *hTERT *ASPSs and splice patterns, a systematic search was set up by establishing panels of cDNA plasmid clones from five different tissues: lung tumour and adjacent tissue, colon tumour, K562 and HL60 cell lines. The cDNA clones were generated by RT-PCR using two primers (p1255 and m3652; Table 1) that produce a 2409-bp fragment from normal full-length *hTERT *mRNA, encompassing the positions of intron 2 to 15 (Fig. 1A). A total of 134 cDNA clones were analysed individually by screening with a panel of primer pairs (Table 1) covering different sub-regions of the *hTERT *cDNA. Clones that contained insertions and/or deletions compared to the normal full-length isoform were analysed by DNA sequencing. The screening detected ten different *hTERT *ASPSs in addition to the normal full-length isoform (Fig. 1B) and six of these have not been described earlier. The donor and acceptor splice sequences used to produce the different ASPSs conform to the GT-AG consensus for intron splicing in eukaryotes [23].

Three novel ASPSs were found that involve insertion of sequences from intron 2. The ASPS named Ins-i2 [1–349] (see Figure 1 for description of the naming convention used) has inserted the first 349 nucleotides of intron 2 and results from the use of an alternative 5'-splice donor sequence within intron 2 (Fig. 1D). The same splice site has also been used to produce the ASPS Ins-i2 [182–349]. But in this ASPS an additional splicing event has removed the first 181 nucleotides of intron 2 by use of the normal 5'-splice donor sequence and an alternative 3'-splice acceptor sequence, leaving nucleotides 182–349 of intron 2 in the spliced product. The third ASPS involving intron 2, Ins-i2 [?–5273], results from the use of an alternative 5'-splice donor sequence within intron 2, but we were not able to characterize this ASPS completely. The RT-PCR cDNA clones containing this ASPS result from internal priming of the 14 3'-most nucleotides of the p1255 primer at position 5212–5225 of intron 2.  Consequently, the sequences located 5' to nucleotide position 5212 of intron 2 were missing from the clones. In principle, the insertion could involve nucleotides 1–5273 of intron 2, or other sub-regions further upstream may be spliced out. The complete lack of Ins-i2 [?–5273] clones produced by normal priming in exon 2 indicates that it either lacks part of exon 2, or is too long to be efficiently amplified by PCR together with other isoforms.

The remaining novel ASPSs contain inserted sequences from intron 14. The ASPSs Ins-i14 [623–705] and Ins-i14 [623–703] both result from a double splicing event in intron 14, which leaves an internal fragment of intron 14 (nucleotides 623–705 and 623–703, respectively) in the spliced product. In addition, two cDNA clones contained an unspliced intron 14.

The screening also detected several ASPSs that have already been described in the literature (see Figure 1B–C). These include Ins-i4 [1–38], Del-e6 [1–36], Del-e7e8 and Ins-i14 [623–781] [9]. Two ASPSs that have been described by others were not observed among the 134 cDNA clones. These include Del-e11 [15] and Ins-i11 [1-?] [9] (see Figure 1B–C). No ASPSs were found that are associated with alternative splicing of intron 3, 5, 9, 10, 11, 12, 13 or 15. Since the primers used to generate RT-PCR cDNA clones anneal in exon 2 and 16 respectively, potential ASPSs involving intron 1 or deletions in exon 1, 2 or 16 could not be addressed in this screening, including Ins-i14 [1–600]/Del-e15e16 [1–492] [14].

### Quantitative analysis of RT-PCR cDNA-clone panels

**Figure 2:**
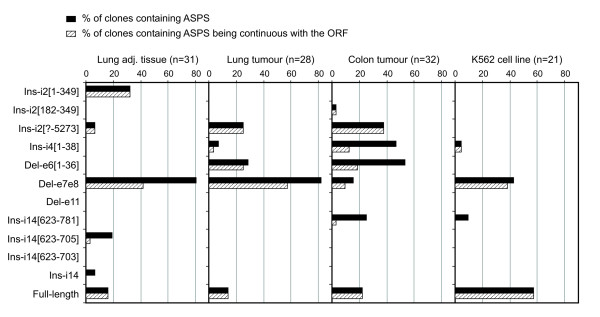
**Frequency of *hTERT *mRNA ASPSs in RT-PCR cDNA clones**. The tissue of origin is indicated above the graph with number of analysed clones in brackets. Black bars show the percentage of clones containing the indicated ASPS. The fraction of these for which the 5'-splice donor site is continuous with the ORF (not masked by other ASPSs further upstream that terminate the ORF) is shown by an adjacent cross-hatched bar.

Assuming that the RT-PCR cDNA clones represent a random selection of mRNA isoforms present in the sample analysed, statistical analyses were employed to address quantitative and correlative relationships. The quantitative data obtained by this approach relates to the percentage-wise distribution of ASPSs within a sample and does not address absolute expression levels. Figure 2 (black bars) summarizes the ASPSs found in RT-PCR cDNA clones from four different samples. Distinct differences were observed in splice pattern between the samples. Besides the normal full-length isoform, Del-e7e8 was the only ASPS detected in all samples and was found in ~80% of clones from lung tumour and adjacent tissue, in 43% from K562 and in 16% from colon tumour. Normal full-length clones were found at moderate levels in colon and lung tumour/adjacent tissue (14–22%) and at a noticeably higher level in clones from K562 (47%). Several ASPSs were found only in clones from lung adjacent tissue. These include Ins-i2 [1–349] (32%), Ins-i14 [623–705] (19%) and Ins-i14 (6%). Other ASPSs were found only in clones from tumour tissues and include Ins-i4 [1–38] (5–47%), Del-e6 [1–36] (0–53%) and Ins-i14 [623–781] (0–25%). The ASPS Ins-i2 [?–5273] was present in a moderate number of clones from lung adjacent tissue (6%) and at considerably higher levels in lung and colon tumour samples (25% and 38% respectively).

Since many ASPSs result in frame shift and premature termination of the ORF, the expected frequency of a particular protein isoform does not depend on the frequency of the corresponding ASPS in mRNA only, but also on the presence of additional ASPSs located further upstream in the same mRNAs. To address this question, the cDNA clones were mapped individually for the presence of alternative 5'-splice donor sites being continuous with the main ORF (Fig. 2, cross-hatched bars). As may be expected, the probability of a particular ASPS being expressed at the protein level depends on its position on the 5' to 3' axis. ASPSs involving intron 2 will always be continuous with the main ORF, while those involving intron 14 have a low probability of becoming part of a protein. Table 2 shows a statistical analysis of correlation computed on all cDNA clones. No strict correlation is apparent between any pair of ASPSs, but two groups show a significant level of co-occurrence. One group consists of Ins-i2 [1–349] and Ins-i14 [623–705]. The other group consists of Ins-i2 [?–5273], Ins-i4 [1–38], Del-e6 [1–36] and Ins-i14 [623–781]. The groups correspond to the distribution of these ASPSs in normal and tumour tissues, respectively.

### Quantitative analysis of cDNA samples

To consolidate the data obtained by screening of RT-PCR cDNA clones, five lung normal/adjacent tissue cDNA samples and seven colon tumour/adjacent tissue cDNA samples were screened by PCR using primer pairs (Table 1) covering smaller sub-regions of *hTERT*. The results (Table 3) confirmed the presence of most ASPSs detected in RT-PCR cDNA clones. The exception was the ASPS Ins-i14 which was not detected in any of the samples. Ins-i14 contains a 781-bp insert and is probably subject to negative selection when co-amplified with considerably shorter products. The ASPS Ins-i14 [1–600]/Del-e15e16 [1–492], which could not be addressed in the analysis of clones, was not detected in any of the samples. Several ASPSs were detected in tissues for which there are no corresponding RT-PCR cDNA clones (see Figure 2). These include Ins-i2 [1–349], Del-e11, Ins-i14 [623–705] and Ins-i14 [623–703] in both lung and colon tumour samples and Ins-i14 [623–781] in lung tumour/adjacent tissues. In addition, two lung adjacent tissue samples contained the ASPSs Ins-i4 [1–38] and Del-e6 [1–36]. Apparently, many ASPSs are more widely expressed than identified by analysis of RT-PCR cDNA clones. This may have several explanations. The different samples used may have different splice patterns (collected from different parts of organ, genetic variation between donors, etc.) and/or the number of mRNA molecules successfully amplified for generation of RT-PCR cDNA clones was low. In addition, statistical sampling errors were expected since the number of clones analysed is limited.

To estimate the quantitative error resulting from co-amplification of PCR products of different sizes, the change in percentages from 35 to 55 cycles of PCR was calculated for individual samples and averaged (Table 3, lower section). Apparently, ASPSs containing long insertions are underestimated, while those having long deletions are overestimated. The most notable case is represented by Del-e11. The PCR product for Del-e11 is 316 bp compared to 505 for normal full-length. The percentage of Del-e11 increased 5-fold from 35 to 55 cycles of PCR, which implies an amplification rate of 1.087 per cycle compared to normal full-length. Extrapolation of quantitative data back to PCR cycle 0 (Table 3, lower section) resulted in Del-e11 percentages close to zero. Two ASPSs, Ins-i4 [1–38] and Del-e6 [1–36], appeared to be over-amplified although they differ little in size from normal full-length. The interpretation of quantitative data for Ins-i2 [?–5273] requires special consideration. Similar to the situation for generating cDNA clones (see above), the PCR product for Ins-i2 [?–5273] is produced by internal priming of the 14 3'-most nucleotides of the p1252 primer within intron 2 at position 5212–5225. The resulting PCR product is shortened by 325 bp compared to normal full-length and is over-amplified with an amplification rate of 1.041. On the other hand, the melting temperature (Tm) for the initial pairing in intron 2 is ~42.5°C, which is 15.5°C below the hybridization temperature used in PCR. Thus the quantitative data (extrapolated in particular) presented for Ins-i2 [?–5273] is most likely underestimated by a considerable amount.

It was apparent from the PCR analysis that cDNA samples from lung tumour yielded more *hTERT *PCR products compared to the other tissues. To provide a rough estimate of these differences, all *hTERT *PCR products produced for each respective tissue were normalized to α-tubulin levels and pooled (Table 3, lower right). The cDNA samples included were limited to those numbered from A through E, which are all pre-normalized with respect to cDNA concentration based on the levels of two housekeeping genes. Compared to lung adjacent tissue, which had the lowest level of *hTERT *products, a 14-fold increase was observed in lung tumour tissue. Colon tumour and adjacent tissues, on the other hand, had very similar *hTERT *levels, about 1.5 to 2-fold higher than in lung adjacent tissue. In a theoretical perspective, these levels are indicative of *hTERT *mRNA abundance (all isoforms included) in the respective tissues.

## Discussion

Regulation of telomerase activity is a complex process involving transcriptional control, post-translational modification, positively and negatively acting factors and alternative splicing [reviewed in [4,24]]. Telomerase activity in cells can be measured directly by TRAP analysis [25]. When addressing telomerase expression by other methods, such as detection of mRNA or protein, caution must be taken to distinguish between functional and non-functional isoforms. In this respect, detailed knowledge about the different *hTERT *isoforms is a prerequisite for proper experimental set-up and correct interpretation of data. By screening a number of RT-PCR-generated cDNA clones from human primary tissues, we identified six novel *hTERT *ASPSs and also four out of six previously described ASPSs. Three novel ASPSs involve alternative splicing of intron 2 and thus have a dominant relationship to other ASPSs located further down-stream in the *hTERT *mRNA. The ASPSs add to the complexity of telomerase regulation and should be taken into consideration when interpreting new and past studies on *hTERT *expression.

In addition to being a tool for detecting sequence differences, the panels of cDNA clones may also be used to address quantitative relationships and mutual correlation of ASPSs in mRNA isoforms. Since the PCR amplicon used to generate RT-PCR cDNA clones is rather large, the differences in size between the different isoforms due to insertions/deletions becomes comparatively smaller. This may promote a more equal amplification rate of the different isoforms being co-amplified in the PCR reaction, compared to PCR covering smaller sub-regions of the *hTERT *cDNA. The situation with over-amplification of the deletion ASPS Del-e11 (Table 3) may serve as an example. Accordingly, Del-e11 was not found among the 134 cDNA clones, which implies that such over-amplification has not occurred when synthesizing the long fragment used for cloning. In this respect, statistical analysis of cDNA clones may represent the most accurate method for quantification of ASPSs in mRNA. The number of clones analysed here (Fig. 2) is not sufficient to meet statistical requirements of sample size. Sampling errors are therefore unavoidable. As seen in the screening of cDNA samples with primer pairs covering smaller sub-regions of *hTERT *(Table 3), several ASPSs were detected in tissues for which there are no corresponding RT-PCR cDNA clones. Thus, many ASPSs appear to be more widely expressed than identified by analysis of clones. The variation in ASPS content observed between cDNA clones and cDNA samples is most likely related to a generally low abundance of *hTERT *mRNA. Some uncertainty also exist regarding the two cDNA clones that contained an unspliced intron 14 (Ins-i14). It is possible that these clones have originated from nuclear mRNA in which intron 14 is retained because of incomplete processing.

A useful consequence of mapping individual cDNA clones is the information obtained on correlation, i.e. the degree by which the different ASPSs tend to occur together in the same mRNAs. This information is necessary for predicting which protein isoforms may be produced in cells since the majority of ASPSs result in frame shift and premature termination of translation (Fig. 1B), which will mask other features located further down-stream in the same mRNA. The RT-PCR cDNA clones were generated from poly(A)^+ ^mRNA and it is reasonable to assume that the corresponding mRNA isoforms are processed by the translation machinery with similar efficiency as the normal full-length isoform. If not, specific mechanisms must exist that can recognize the different internal insertions/deletions in these mRNAs and suppress transport/translation. Thus, the expected frequency of a particular protein isoform (frame shift, insertion or deletion of aa) depends in part on the frequency of the corresponding ASPS in mRNA, but also on the presence of additional ASPSs located further upstream. Since the majority of *hTERT *transcripts in lung and colon tissues contained two or more ASPSs, ASPSs located downstream (and in particular 3' to exon 8) are rarely continuous with the ORF (Fig. 2). Instead, the pool of hTERT protein in human lung and colon tissues seems to be dominated by truncated isoforms corresponding to the ASPSs Ins-i2 [1–349], Ins-i2 [?–5273] and Del-e7e8.

The correlation analysis (Table 2) defined two groups of ASPSs with a tendency to occur together in the same mRNAs, but no pair of ASPSs showed a strict positive correlation. With regard to negative correlation the results were less conclusive, since some ASPSs occur at a low frequency. However, for the more abundant ASPSs being present in the same tissue samples (Ins-i2 [?–5273], Ins-i4 [1–38], Del-e6 [1–36], Del-e7e8), any single ASPS were found both combined with and separate from other ASPSs in individual cDNA clones, which implies that the alternative splicing occurring at these sites are more or less independent events. If no ASPSs are strictly correlated, the *hTERT *gene may give rise to as many as 384 different *hTERT *mRNA isoforms. Many of these are similar in size to normal full-length *hTERT*. Considering the number and location of in-frame insertion/deletion ASPSs (Fig. 1B) and the combination of these with the other ASPSs, the 384 mRNA isoforms may give rise to 64 different hTERT protein isoforms. As explained above, the abundance of the different protein isoforms will vary greatly according to ASPS frequency and position on the 5' to 3' axis.

RT-PCR analysis of a sub-region in *hTERT *containing the Del-e6 [1–36] and Del-e7e8 deletion splice sites is often used to estimate telomerase expression, since mRNA lacking this alternative splicing is considered to be normal full-length. Our data showing that a large percentage of *hTERT *mRNA may be alternatively spliced in intron 2 implies that this analysis is insufficient for estimation of *hTERT *isoforms. The increased complexity in telomerase expression necessitates a more detailed analysis of *hTERT *isoforms, which can measure both upstream and downstream splicing at the single-transcript level. Analysing the different sub-regions in separate PCRs (Table 3) fails to detect whether the different ASPSs are present in the same transcripts or not (correlation), which is required for estimation of normal full-length *hTERT*. Direct analysis of a PCR product covering all ASPSs is also problematic, since many isoforms (different combinations of ASPSs) are similar in size to normal full-length. It would require a method with perfect one-nucleotide resolution for fragments above 2 kbp and an acceptable quantitative read-out. One solution to these problems is to map long PCR products (or cDNA clones) covering all ASPSs on individual basis as shown in Figure 2, which gives a direct measurement of all different isoforms being present.

## Conclusion

Previous work addressing identification of *hTERT *ASPSs [9,14,15] have focused on cell lines, which normally contain high telomerase activity and a high percentage of normal full-length *hTERT *mRNA. In the present study, by analysing a modest number of RT-PCR-generated cDNA clones from two different human tissues, six novel ASPSs were identified of which two represented dominating hTERT isoforms. A better understanding of *hTERT *mRNA processing and its implications for telomerase regulation will require more studies on human primary tissues, including those that are low or negative for telomerase activity. It is also necessary to use methods that do not discriminate between mRNA isoforms based on size, as opposed to PCR, since ASPSs may involve intronic insertions of considerable length. Detailed knowledge of the different hTERT isoforms present in human tissues and the ability to distinguish between them is required to obtain a proper understanding of telomerase function and regulation, and may identify hTERT sequences suitable for targeted cancer immuno therapy.

## Methods

### Tissue samples and cDNA synthesis

Lung and colon tumour/adjacent tissue matched pair cDNA samples A-E were obtained from Clontech (Catalog #: K1433-1, K1434-1). Additional tissue samples were removed by surgery (The Norwegian Radium Hospital, Oslo, Norway) and stored in RNAlater (Sigma-Aldrich) for preservation of RNA. The samples were homogenized in Trizol Reagent (Invitrogen, Basel, Switzerland) using the Ultra-Turrax T8 homogenizer (IKA-Werke, Staufen, Germany) and total RNA was isolated and purified according to the Trizol Reagent manual. Poly(A)^+ ^mRNA was isolated and double-selected from total RNA using the GenoPrep Direct mRNA kit (GenoVision, Oslo, Norway) and cDNA synthesis was performed by using the StrataScript Reverse Transcriptase (Stratagene, La Jolla, CA) with oligo(dT) priming as described by the manufacturer.

### Generation of hTERT RT-PCR cDNA clones

PCR fragments encompassing intron positions 2–15 in the *hTERT *gene was produced in two consecutive PCR reactions using two primers (p1255 and m3652; Table 1) that hybridize within exon 2 and 16, respectively. The first PCR was performed using the HotStarTaq kit (Qiagen) with 10% vol/vol cDNA-synthesis reaction as template, 2% DMSO, 30 cycles of PCR, annealing at 58°C and extension for 6 minutes. The second PCR was run using the Pfu Turbo kit (Stratagene) with 10% vol/vol of the first PCR as template and other modifications as described for the first PCR. The product was then separated in an agarose gel and a segment containing PCR products in the range 1 to 10 kb was cut out and purified using the Wizard PCR Preps DNA Purification System (Promega). The PCR products were digested with *EcoR *I and *Xba *I and ligated between the same sites in the *pBluescript SK+ *vector (Stratagene). Following transfection of E. coli DH10B and growth on LA-amp, colonies were picked at random for production of plasmid DNA (Wizard Plus SV Minipreps, Promega).

### Mapping of hTERT RT-PCR cDNA clones

Individual *hTERT *RT-PCR plasmid clones were screened for the presence of ASPSs by running PCR with a panel of 6-fam-labelled primer pairs (Table 1) covering different regions of the *hTERT *cDNA. The products were analysed with the MegaBACE 1000 capillary electrophoresis unit (Amersham Biosciences). Clones that contained insertions and/or deletions compared to normal full-length *hTERT *cDNA were sequenced through the relevant region using the MegaBACE sequencing system (Amersham).

### PCR analysis of cDNA samples

cDNA samples were screened for the presence of *hTERT *ASPSs by performing two consecutive PCR reactions with a panel of 6-fam-labelled primer pairs (Table 1) covering different regions of the *hTERT *gene. The first PCR was run using the HotStarTaq kit with 10% vol/vol cDNA-synthesis reaction as template, 35 cycles of PCR, annealing at 58°C and extension for 2 minutes. The second PCR was run using the Pfu Turbo kit (Stratagene) with 10% vol/vol of the first PCR as template, 20 cycles of PCR and other parameters as described for the first PCR. The products were analysed with the MegaBACE 1000 capillary electrophoresis unit.

## Authors' contributions

SSL carried out the design of the study, most of the experiments and drafted the manuscript. EF carried out molecular biology studies. GG participated in the coordination and revising of the manuscript. All authors read and approved the final manuscript.

## Figures and Tables

**Table 1:** Primer pairs used for *hTERT *PCR analysis

Target ^1)^	Primer pair ^2)^	Ampl. (bp)
intron 2–15	p1255: ggaattctggagctgcttgggaacca m3652: cgtctagagccggacactcagccttca	2409
intron 2	p1252: tgtttctggagctgcttgggaacca m1725: tcatcagccagtgcaggaacttg	474
intron 2–3	p1509: tggggctccaggcacaacgaa m1955: cttggggatgaagcggagtct	447
intron 4	p1870: tgcgggagctgtcggaagcagag m2170: cgcacacgcagcacgaaggt	301
intron 5–9	p2182: cgccgcctgagctgtactttgtc m2681: gctctagatccaccaaacgcaggagca	507
intron 10–13	p2660: gctgctcctgcgtttggtggatgat m3164: ggggttcttccaaacttgctgatg	505
intron 14–15	p3194: ggcctccctctgctactccatcct m3652: cgtctagagccggacactcagccttca	466
intron 14–15	p3194: ggcctccctctgctactccatcct m3925: gggcacacctttggtcactcc	732

^1) ^Positions of indicated introns are included in PCR product. ^2) ^Primer sequences are from 5' to 3'. p, forward primer; m, reverse primer.

**Table 2:** Statistical correlation between *hTERT *ASPSs ^1)^

	Ins-i2 [1–349]	Ins-i2 [182–349]	Ins-i2 [?–5273]	Ins-i4 [1–38]	Del-e6 [1–36]	Del-e7e8
Ins-i4 [1–38]	-0.12	-0.04	0.51			
Del-e6 [1–36]	-0.14	-0.04	0.32	0.34		
Del-e7e8	0.28	-0.09	-0.03	-0.22	-0.10	
Ins-i14 [623–781]	-0.08	-0.02	0.42	0.44	0.30	-0.12
Ins-i14 [623–705]	0.63	-0.02	-0.09	-0.09	-0.10	0.14
Ins-i14 [623–703]	-0.02	-0.01	-0.04	-0.04	-0.04	-0.09
Ins-i14	-0.03	-0.01	-0.05	-0.05	-0.06	0.12

^1) ^Correlation coefficient calculated according to the formula: ρ_X, Y _= cov(X, Y)/(σ_X_·σ_Y_), where σ_x_^2 ^= Σ(X_i_-μ_x_)^2^/n, σ_y_^2 ^= Σ(Y_i_-μ_y_)^2^/n (ρ; correlation coefficient, cov; co-variance, σ; standard deviation). The correlation coefficient is a number between -1 and 1: -1, mutually exclusive (all clones contain one or the other); 0, random distribution or both not present; 1, always occur together (independently of frequency).

**Table 3:** PCR analysis of *hTERT *ASPSs in lung and colon tumour/adjacent tissue cDNA samples

Tissue	Sample ^1)^	Ins-i2 [1–349]	Ins-i2 [182–349]	Ins-i2 [?–5273]	Ins-i4 [1–38]	Del-e6 [1–36]	Del- e7e8	Del-e11	Ins-i14 [623–781]	Ins-i14 [623–705]	Ins-i14 [623–703]	α-tub ^2)^
Lung	A	25.2	0.0	*7.3	5.6	7.3	71.5	0.0	8.5	0.0	0.0	77.1
Adjacent	B	N	N	N	0.0	0.0	99.9	0.0	0.0	0.0	0.0	70.6
	C	0.0	0.0	0.0	0.0	0.0	0.0	0.0	47.5	0.0	0.0	61.9
	D	N	N	N	0.0	0.0	*100.0	N	0.0	0.0	0.0	62.5
	E	0.0	0.0	0.0	100.0	100.0	100.0	N	*51.3	0.0	0.0	61.1
Lung	A	14.1	*0.5	13.3	1.8	2.9	39.5	0.0	1.4	0.4	*0.3	73.2
Tumour	B	58.1	0.0	0.0	0.0	0.0	54.4	0.0	31.7	9.7	0.0	69.9
	C	10.7	0.0	9.1	5.4	*1.8	76.9	*12.7	10.6	*0.3	0.0	73.2
	D	0.0	0.0	0.0	0.0	0.0	76.7	0.0	18.5	0.0	0.0	79.3
	E	7.0	*0.1	*42.8	6.3	6.2	82.6	5.4	8.5	0.8	0.9	68.4
Colon	A	0.0	0.0	0.0	*24.2	14.8	75.3	0.0	0.0	0.0	*2.9	53.7
Adjacent	B	0.0	0.0	0.0	0.0	8.8	64.5	0.0	*3.6	0.0	0.0	64.4
	C	0.0	0.0	0.0	0.0	*4.7	65.7	0.0	10.2	0.0	0.0	55.9
	D	0.0	0.0	0.0	0.0	0.0	53.4	0.0	2.7	0.0	0.0	45.6
	E	0.0	0.0	0.0	0.0	0.0	81.0	0.0	*0.3	0.0	7.3	54.4
	F	0.0	*100.0	0.0	N	0.0	57.4	0.0	0.0	0.0	0.0	82.4
	G	*0.1	*0.4	50.0	20.4	7.3	57.1	*0.6	3.1	0.0	0.0	88.9
Colon	A	0.0	0.0	*100.0	0.0	0.0	69.7	0.0	0.0	0.0	0.0	57.3
Tumour	B	29.2	0.0	0.0	19.6	22.3	63.2	0.0	16.6	0.0	*2.0	70.2
	C	0.0	0.0	0.0	0.0	0.0	76.6	0.0	40.2	0.0	0.0	67.8
	D	0.0	0.0	0.0	0.0	0.0	*54.1	0.0	13.2	0.0	0.0	68.3
	E	*6.2	0.0	0.0	8.8	0.0	81.5	0.0	8.0	0.0	0.0	60.1
	F	2.5	0.0	20.2	22.7	7.1	58.1	0.4	3.9	0.7	0.7	91.7
	G	0.0	0.0	94.9	4.2	5.3	53.2	0.0	4.5	0.5	0.7	90.8
Pooled ^3)^	Tissue											hTERT ^4)^sum total
PCR x35	Lung adjacent	25.1	0.0	0.0	11.1	17.9	61.9	0.0	13.6	0.0	0.0	1.0
	Lung tumour	11.9	0.0	8.8	3.3	3.2	56.3	1.9	5.5	0.6	0.2	14.2
	Colon adjacent	0.0	0.0	37.6	8.0	5.1	64.5	0.0	3.0	0.0	0.7	1.8
	Colon tumour	6.7	0.0	69.6	6.4	5.3	55.0	0.2	4.8	0.6	0.7	1.5
Extrapolated												
Δ 55/35 ^5)^		0.55	-	2.22	1.71	2.02	1.11	5.29	0.76	1.03	1.06	
Ampl. rate ^6)^		0.970	-	1.041	1.027	1.036	1.005	1.087	0.986	1.002	1.003	
PCR start ^7)^	Lung adjacent	72.0	-	0.0	4.3	5.2	51.8	0.0	22.0	0.0	0.0	
	Lung tumour	34.1	-	2.2	1.3	0.9	47.1	0.1	8.9	0.6	0.2	
	Colon adjacent	0.0	-	9.3	3.1	1.5	53.9	0.0	4.9	0.0	0.7	
	Colon tumour	19.2	-	17.2	2.5	1.5	46.1	0.0	7.8	0.5	0.6	

^1) ^Values are percentage ASPS of the total *hTERT *PCR products produced in each respective PCR reaction. ASPS products detected after 55 cycles of PCR only are denoted with an asterisk; remaining values are from 35 cycles of PCR. N; no PCR products (including normal full-length) detected after 35 or 55 cycles of PCR. ^2)^α-tubulin values are percentage of primer incorporated in product after 35 cycles of PCR. ^3) ^For calculation of pooled data the samples were normalized to α-tubulin levels and PCR products produced after 35 cycles of PCR were combined before calculation of percentages (group of samples treated as one sample). ^4) ^Sum totals (relative values) of all *hTERT *PCR products produced for a given tissue calculated from normalized values. ^5) ^Average change in percentages from 35 to 55 cycles of PCR computed from individual samples; the number is a factor where 1 means no change. ^6) ^Amplification rate per cycle compared to normal full-length product calculated by taking the 20^th ^root of Δ. ^7) ^Estimation of ASPS percentages by extrapolation of data from 35 cycles of PCR using the formula: (ASPS %)/(Ampl. rate)^35^.
